# No Evidence for Distinct Transcriptomic Subgroups of Devil Facial Tumor Disease (DFTD)

**DOI:** 10.1111/eva.70091

**Published:** 2025-04-01

**Authors:** Cleopatra Petrohilos, Emma Peel, Kimberley C. Batley, Samantha Fox, Carolyn J. Hogg, Katherine Belov

**Affiliations:** ^1^ School of Life and Environmental Sciences The University of Sydney Sydney New South Wales Australia; ^2^ Australian Research Council Centre of Excellence for Innovations in Peptide & Protein Science The University of Sydney Sydney New South Wales Australia; ^3^ Save the Tasmanian Devil Program Department of Natural Resources and Environment Hobart Tasmania Australia

**Keywords:** contagious cancer, unsupervised clustering, wildlife disease

## Abstract

Contagious cancers represent one of the least understood types of infections in wildlife. Devil Facial Tumor Disease (comprised of two different contagious cancers, DFT1 and DFT2) has led to an 80% decline in the Tasmanian devil ( *Sarcophilus harrisii* ) population at the regional level since it was first observed in 1996. There are currently no treatment options for the disease, and research efforts are focused on vaccine development. Although DFT1 is clonal, phylogenomic studies have identified different genetic variants of the pathogen. We postulated that different genetic strains may have different gene expression profiles and would therefore require different vaccine components. Here, we aimed to test this hypothesis by applying two types of unsupervised clustering (hierarchical and k‐means) to 35 DFT1 transcriptomes selected from the disease's four major phylogenetic clades. The two algorithms produced conflicting results, and there was low support for either method individually. Validation metrics, such as the Gap statistic method, the Elbow method, and the Silhouette method, were ambiguous, contradictory, or indicated that our dataset only consisted of a single cluster. Collectively, our results show that the different phylogenetic clades of DFT1 all have similar gene expression profiles. Previous studies have suggested that transcriptomic differences exist between tumours from different locations. However, our study differs in that it considers both tumor purity and genotypic clade when analysing differences between DFTD biopsies. These results have important implications for therapeutic development, as they indicate that a single vaccine or treatment approach has the potential to be effective for a large cross‐section of DFT1 tumors. As one of the largest studies to use transcriptomics to investigate phenotypic variation within a single contagious cancer, it also provides novel insight into this unique group of diseases.

## 1 Introduction

As the biodiversity and climate crises deepen, infectious wildlife diseases are becoming more prevalent (El‐Sayed and Kamel 2020). One of the rarest and least understood modalities of infection is contagious cancer, tumor cells that can be transmitted from one individual to another (Metzger and Goff 2016). To date, transmissible cancers have only been documented in 11 species (two vertebrates and nine invertebrates), although the true number is estimated to be much higher (Metzger and Goff 2016; Dujon et al. 2021; Hart et al. 2023; Bruzos et al. 2023; Metzger et al. 2016; Hammel et al. 2022; Hallmann et al. 2022; Santamarina et al. 2024; Yonemitsu et al. 2023).

Contagious cancers have devastating impacts on the hosts' ecological function and commercial industries such as aquaculture (Dujon et al. 2020). A contagious cancer that is currently of particular conservation concern is Devil Facial Tumor Disease (DFTD). This disease affects the largest marsupial carnivore, the Tasmanian devil ( *Sarcophilus harrisii* ), and has caused population crashes of up to 80% across the species' range (Lazenby et al. 2018). There are now two forms of DFTD: DFT1 that first arose in the 1990s in the northeast of Tasmania (Loh et al. 2006), and DFT2 that was first detected in the southeast of Tasmania in 2014 (Pye, Pemberton, et al. 2016). DFT1 has spread across the island state of Tasmania, while DFT2 remains restricted to the southeast of Tasmania (Lazenby et al. 2018; James et al. 2019). Here we focus on DFT1.

DFT1 is almost always fatal, with very few regressions of the disease documented (Wright et al. 2017; Margres et al. 2020). A genome‐wide association analysis indicated that regression may be caused by variants near the *PAX3* gene that disrupt angiogenesis to tumors (Wright et al. 2017), while a comparative genomics approach also identified a mutation in the putative tumor suppressor *RASL11A* (Margres et al. 2020). There are currently no treatments for either form of DFTD, although candidate therapeutics have been tested in vitro (Petrohilos et al. 2023; Patchett et al. 2018; Fernandez‐Rojo et al. 2018; Stammnitz et al. 2018; Kosack et al. 2019) and in murine models for DFT1 (Ikonomopoulou et al. 2021). There are also research efforts focused on developing a vaccine (Tovar et al. 2017; Pye et al. 2018, 2021; Flies et al. 2020; Kayigwe et al. 2022) A number of immunostimulatory adjuvants have been identified (Pye et al. 2018; Patchett et al. 2017) that may be used in combination with recombinant DFT proteins to trigger an immune response (Flies et al. 2020). The aim is to deliver this via an oral bait vaccine platform similar to the successful rabies vaccine (Flies et al. 2020), with trials having shown that both captive and wild devils will consume placebo baits (Dempsey et al. 2022).

Cancer—even tumors that affect the same tissue type—is an umbrella term rather than a single disease. For this reason, classifying tumors into subtypes based on molecular differences is important for therapeutic development and maximizing treatment efficacy (Zhao et al. 2020; Collisson et al. 2019). However, transmissible cancers are unique in this respect. Contagious cancers are clonal and so do represent a single disease. Previous studies have observed that DFT1 is constantly evolving yet remains remarkably stable for a cancer (Deakin and Belov 2012; Murchison et al. 2012; Deakin et al. 2012; Ingles and Deakin 2015; Kwon et al. 2020). Although it has a highly rearranged genome, additional mutations such as translocations and aneusomy are rare in primary tumors (Pearse et al. 2012). Interestingly, these mutations are common in metastases and long‐term cell culture of DFT1, which may indicate a strong selective pressure in vivo (Pearse et al. 2012). This may indicate that mutations exceeding a particular threshold result in cells that are either not viable or not transmissible (Pearse et al. 2012). The one exception to this is tetraploidy, with whole genome duplications occurring multiple times in the disease's history (Kwon et al. 2020). Polyploidy is likely advantageous for DFT1 as it can mask Muller's ratchet—the accumulation of deleterious mutations in asexual organisms that leads to genomic erosion (Ujvari et al. 2014).

Early evidence suggested four distinct karyotypic strains of DFT1 (Deakin et al. 2012), categorized by the presence or absence of five “marker” chromosomes (Marker 1—Marker 5) (Deakin et al. 2012). One study suggested the strains had differential growth rates in vitro; however, this conclusion was based on a low number of replicates (*n* = 1–3 among the four strains) (Pearse et al. 2012). Subsequent studies have since shown minimal microsatellite (Pearse et al. 2012), epigenetic (Ingles and Deakin 2015; Ujvari et al. 2013) or cytogenetic (Deakin et al. 2012) differences between the four strains. In 2020, Kwon et al. showed that the marker characterizing strains two, three, and four (Marker 5) is highly unstable and has been lost at least 27 times between 2003 and 2018 (Kwon et al. 2020). This suggests that classifying tumors into these four strains may not be a biologically meaningful way to categorize the disease due to the unreliability of observing the defining markers.

More recently, phylogenomic methods have identified six phylogenetic clades (A1, A2, B, C, D, and E) of DFT1, although the latter two (D and E) have failed to persist in the wild, and clade A1 has not been detected since 2012 (Kwon et al. 2020; Stammnitz et al. 2023). There does not appear to be any association between phylogenetic clades and karyotypic strains. Kwon et al. (2020). used a robust combination of mitochondrial variants, nuclear variants, and copy number variants to build one of the largest tumor phylogenies to date (over 600 genomes collected between 2003 and 2018). Although such phylogenomic methods are useful for tracing the evolutionary trajectory of the disease, genetic differences may not necessarily translate to functional differences. Many variants may be effectively neutral; for example, those that are synonymous, intronic, or in genes that are not expressed. Instead, phenotypic differences between tumor subtypes can be characterized using transcriptomics. RNAseq data is closely linked with phenotype (Guinney et al. 2015) as it only measures the genes that are expressed within a sample. Unlike genomic variation, transcriptomic differences do equate to functional differences. RNAseq data has been used to identify potential antigens for immunotherapy in a range of human cancers (Wu, Duan, et al. 2022; Wu, Qin, et al. 2022) and transcriptomic assays such as MammaPrint, Oncotype DX, and PAM50 are frequently used to guide the treatment of breast cancer (Chaudhuri et al. 2021). In the case of DFT1, the number of transcriptomic subtypes may influence how many neoantigens must be targeted by a vaccine. It may also aid in conservation management decisions, such as translocating diseased animals to reduce the spread of different strains.

RNAseq has been used to investigate phenotypic variation within DFT1. One study investigated transcriptomic differences in tumors from different geographic regions (Kozakiewicz et al. 2021) but did not seek to identify molecular subtypes. The dataset was also limited to 19 samples from three locations. Studies have also raised concerns about the effectiveness of a single DFT1 vaccine due to the potential heterogeneity of the disease (Pearse et al. 2012). Here, we generated a much larger RNAseq dataset: 35 DFT1 samples from 12 locations across central Tasmania where DFT1 has been present since 2003. These samples represent all four phylogenomic clades and karyotypic strains (Kwon et al. 2020). Our aim was to determine if DFT1 has distinct patterns of gene expression that categorize tumors into different subgroups, and if so, do these align with those noted in the phylogenomic studies to inform vaccine and therapeutic treatment development.

## 2 Methods

### 2.1 Data Collection

Biopsies from DFT1 primary tumors were collected by the Save the Tasmanian Devil Program between 2006 and 2015 from multiple sites across Tasmania as part of their annual monitoring program and shared with us for the purposes of this study (Table S1). The biopsies selected for this study were from tumors that had previously been genotyped and assigned to a phylogenetic clade, as well as to a karyotypic strain (Kwon et al. 2020). They included representative samples from each of the four major clades: 11 samples from clade A1, 10 from clade A2, 10 from clade B, and four from clade C (the smallest of the four major clades). Samples from clades D and E were not included in our study as these clades have failed to persist in the wild (Kwon et al. 2020). Our dataset also contained representatives from all major karyotypic strains (1–4).

Total RNA was extracted from DFT1 biopsies using a Qiagen RNeasy mini kit (Qiagen, Cat. No. 74104). RNA quality was assessed using the RNA nano 6000 kit on the Bioanalyzer (Agilent) and samples with an RNA integrity score (RIN) greater than seven were submitted to Ramaciotti Centre for Genomics (The University of New South Wales) for sequencing. All samples underwent TruSeq stranded mRNA library prep (Illumina) and were sequenced as paired‐end 150 bp reads across an SP flowcell on the Illumina NovaSeq6000. This resulted in 50–107 million read pairs per sample.

As a control for comparison, raw RNAseq reads from Tasmanian devil healthy tissue and DFT1 biopsies were downloaded from NCBI (BioProject PRJEB34650 [Stammnitz et al. 2023] and PRJEB28680 [Patchett et al. 2020]). The details of the samples downloaded are in File S1A.

### 2.2 Data Analysis

All samples were quality assessed using FastQC v0.11.8 (Andrews 2010) and trimmed of low quality and adaptor sequences using Trimmomatic v0.39 (Bolger et al. 2014). Reads were then aligned to the Tasmanian devil reference genome mSarHar1.11 (NCBI: GCF_902635505.1) (Stammnitz et al. 2023) using STAR v2.7.8a (Dobin et al. 2013). Default parameters were used for the DFT1 reads from this study and the samples from Patchett et al. (2020). As the samples from Stammnitz et al. (2023) had a much shorter read length (75 bp) than those generated in this study (150 bp), we adjusted the alignment parameters to improve the mapping rate and make the samples comparable (‐sjdbOverhang 74 ‐outFilterScoreMinOverLread 0.1 ‐outFilterMatchNminOverLread 0.1).

Some of the reads downloaded from NCBI had very low alignment rates (File S1A) which may indicate poor sequencing accuracy or DNA contamination (Conesa et al. 2016). For this reason, only data that consisted of at least two biological replicates with at least 75% uniquely mapped reads was retained for further analysis. This resulting dataset consisted of 54 samples from nine different tissues, including 37 DFT1 samples (including the 35 generated here and two from Patchett et al. 2020), two axillary nerve (Stammnitz et al. 2023), two bone marrow (Stammnitz et al. 2023), two brain (Patchett et al. 2020), two cerebellum (Stammnitz et al. 2023), two cerebrum (Stammnitz et al. 2023), two spleen (Patchett et al. 2020), three testes (Stammnitz et al. 2023; Patchett et al. 2020) and two trigeminal nerve (Stammnitz et al. 2023).

Alignments were summarized into gene counts using featureCounts in the subread package v1.5.1 (Liao et al. 2014). Gene counts were then input into R v4.1.3 (R Development Core Team 2022). As the samples in Patchett et al. (2020) consisted of technical replicates, these were summed prior to further analysis. Lowly expressed genes with a total count less than 50 across all samples were excluded from the analysis. Normalization factors were calculated using trimmed mean of M values (TMM) to account for differences in raw library sizes (edgeR v3.36.0, Robinson et al. 2010).

To ensure that the different methods of sequencing used in the different studies did not bias the results, a redundancy analysis (RDA) was performed using the vegan package v 2.6–6.1 (Oksanen et al. 2018) in R. Gene counts (normalized to log counts per million) were used as the response variable, and tissue type and study were both used as the predictor variables in the model. The significance of each term was tested using the anova.cca function in vegan with 999 permutations. Variance partitioning was then conducted using the varpart function in vegan to assess the percentage of variance explained by each response variable (i.e., tissue type and study). The significance of the variance partitioning was also assessed using anova.cca with 999 permutations.

Multidimensional scaling (MDS) was used to check variation across the samples (limma v3.50.3 [Ritchie et al. 2015]). Four DFT1 samples were identified as outliers using two different methods (MDS and hierarchical clustering) (Figures S2 and S3). These four samples clustered more closely to the spleen samples (*N* = 2) on the first dimension and the bone marrow samples (*N* = 2) on the second dimension than did other DFT1 samples (Figure S4). Both spleen and bone marrow are tissues that contain a high proportion of immune cells, so these results suggest the section of the biopsy from which RNA was extracted contained a higher proportion of immune cells than DFT1 cells. For this reason, these four outliers were excluded from subsequent analysis.

### 2.3 Purity Estimation

Tumor purity was further estimated by using the somatic substitution variant allele fraction (VAF) distributions of each sample, as used previously for DFTD (Kwon et al. 2020; Stammnitz et al. 2023). VAF is the proportion of reads covering a particular variant and is calculated by *n*_*s*_/*N*_*s*_ (where *n*_*s*_ is the number of reads containing the variant and *N*_*s*_ is the total number of reads) (Dentro et al. 2021). VAF reflects the zygosity of a locus: homozygous reference loci should have a VAF around 0, heterozygous loci around 0.5, and homozygous variant loci around 1 (Strom 2016). As most mutations in DFT1 exist in a heterozygous state, the average VAF of a clonal population of DFT1 cells is expected to be 0.50 (Stammnitz et al. 2023). For this reason, *VAF*_*HET*_ (defined as either the mode or median VAF of all heterozygous variants in a sample) has been used to measure the purity of DFT1 samples in previous studies (Kwon et al. 2020; Stammnitz et al. 2023).

First, samples were preprocessed with Opossum (Oikkonen and Lise 2017). Many variant callers do not perform optimally when applied directly to RNAseq data, as splice junctions cause the reads to be split and lose information (Oikkonen and Lise 2017). Opossum modifies the reads prior to splitting to ensure that information is retained and improve variant calling sensitivity. We then called variants using Platypus v0.1.5 (Rimmer et al. 2014) with settings minPosterior = 0, minBaseQual = 30, badReadsThreshold = 30, badReadsWindow = 15, minFlank = 0, and minReads = 1. As Platypus does not work on chromosomes longer than 536 Mb and chromosomes 1, 2, and 3 of the reference genome (NCBI GCF_902635505.1) are 611 to 716 Mb, we split these chromosomes into 500 Mb windows prior to variant calling.

Single nucleotide variants (SNVs) were extracted, and any variants flagged as badReads, sb (strand bias), MMLQ (median minimum base quality for bases around variant) < 30, and QUAL < 20 were excluded. We also excluded any variants within 5 bp from a simple repeat region (as annotated by TandemRepeatsFinder v4.09.1 [Benson 1999]), within 500 bp from the contig start/end, or within 1000 bp of the scaffold start/end.

Variants were considered to be somatic if they were one of the 1311 “trunk” variants identified by Stammnitz et al. (2023). Of these 1311 mutations, 421 occur in exonic regions. These “trunk” variants are present in all DFT1 tumors but are absent from all healthy Tasmanian devil genomes examined so far. We classified a variant as “present” in our samples if it was supported by > 3 reads.

For each sample, we calculated the VAF of each variant by dividing TR (Total reads supporting the variant) by TC (Total coverage at the locus). For example, if there were 3 reads supporting a particular SNV and 5 total reads at that locus, the VAF of that SNV would be 0.6. We plotted the VAF distribution for each sample and defined *VAF*_*HET*_ as the maximum density of the heterozygous peak similarly to Stammnitz et al. (2023). An example distribution plot is included in Figure S1. We then estimated each sample's purity (ρ) using the $\rho =2*{\mathrm{VAF}}_{\mathrm{Het}}$ formula that has been used by Kwon et al. (2020) and Stammnitz et al. (2023). Distribution plots for all samples are included in Data S2.

While this method for estimating tumor purity has its limitations due to the low number of trunk variants in expressed genes, it represents the best available method due to the nature of the data. Ideally, single‐cell RNA sequencing would be used to accurately assess purity by generating gene expression profiles for tumor and multiple host cell types to assign cell populations. However, single‐cell RNAseq data is not available for DFTD or Tasmanian devils, and the logistics of collecting biopsies from wild animals currently render such a method difficult at this time.

Only samples with a purity > 80% were retained for further analysis (*n* = 27). This threshold was chosen as it represented a balance between maximizing accuracy and maximizing sample size. Purity estimates for all samples are included in File S1B. For each sample, the number of reads supporting the reference and alternative alleles for each variant is included in File S1C.

For comparison, the analysis was also run separately on the full dataset (excluding the four outliers).

### 2.4 Unsupervised Clustering

Upper quartile normalization was applied to account for differences across sequencing lanes (EDAseq v2.28.0 [Risso et al. 2011]) and transcripts per million (TPM) were calculated to account for differences in gene lengths (scater v1.22.0 [McCarthy et al. 2017]). Counts were then log‐transformed for variance stabilization across the samples.

Unsupervised clustering encompasses a variety of machine learning algorithms that reveal meaningful groups in unlabeled data (Dalmaijer et al. 2022). They are commonly used to identify biologically relevant subsets of data such as cancer subtypes (Guinney et al. 2015; Tan et al. 2011; Lei et al. 2013; Oh et al. 2018; Sekiguchi et al. 2020; Lapointe et al. 2004; Robertson et al. 2020). One caveat of these methods is that they will identify clusters, regardless of whether they exist, so it is critical to test the robustness of the resulting subsets (Adolfsson et al. 2019). Common methods include: data visualization (Tan et al. 2011), using multiple algorithms to identify a consensus of subtypes (Guinney et al. 2015; Lei et al. 2013) and clustering validation indices (Xiong et al. 2018).

Here, we used two methods of dimension reduction for preliminary data visualization: MDS and t‐Distributed Stochastic Neighbor Embedding (t‐SNE) (van der Maaten and Hinton 2008). t‐SNE was performed using Rtsne v.0.16 with 30 initial principal components and a perplexity (a hyperparameter that reflects the density of the data) of 9. This was run 500 times, and the optimal run was identified as the one with the lowest KL‐divergence. We used heatmaps to visualize potential patterns in the data. We first used the 1000 most variable genes (those with the highest standard deviation), then iteratively halved this number to use progressively fewer genes (500, 250, and 100). This was to test for a genetic signal driven by a small number of genes that may be lost in the noise of the entire dataset.

The 1000 most variable genes were then used as input to two different clustering algorithms: hierarchical clustering (using Euclidean distance and Ward's D) and k‐means clustering (Figure S2). For each method, the optimum number of clusters in the data was determined using three different cluster validation metrics in factoextra v1.0.7 (Kassambara and Mundt 2017): Silhouette method (Rousseeuw 1987), elbow method, and Gap statistic method (Tibshirani et al. 2001). *K* = 4 was chosen for k‐means clustering based on the results of the Silhouette method (see Section 3).

The hierarchical clustering results were then further tested using two methods: pvclust v2.2–0 (Suzuki and Shimodaira 2006) was used for bootstrap analysis using 10,000 bootstraps, and sigclust2 v1.2.4 (Kimes et al. 2017) was used to perform a Monte Carlo simulation‐based significance testing.

The results from the two clustering methods (hierarchical and k‐means) were compared visually using the R package dendextend 1.17.1 (Galili 2015).

## 3 Results

Over 97% of all reads were retained following trimming, with a mean of 78 million reads per sample (range: 13–142 million reads). The reads generated in this study had high rates of uniquely mapped reads (75%–86%). The full list of the number of reads and mapping statistics for each sample is included in File S1A.

The RDA and variance partitioning analyses indicated that different sequencing methods did not significantly influence the estimation of gene counts (Table S2). While tissue type was significant as a predictor variable (*p* = 0.001), the study was not (*p* = 0.2). This shows that sequencing type does not introduce any bias in gene expression data, and combining data from multiple studies is an appropriate method for maximizing sample size.

Exploratory analysis showed a difference in gene expression between DFT1 and healthy tissue, with DFT1 samples largely clustering together in the MDS plot, and minimal differences between the various types of healthy tissue biopsies, including those that were sequenced in different studies. This was achieved after four outliers (Figures S2 and S3) and other samples with purity < 80% were removed from the DFT1 dataset (Figure S4). In particular, the four main outliers clustered most closely with the spleen and bone marrow samples. As both spleen and bone marrow are haemopoietic organs that contain immune cells, we hypothesize that the sections of the biopsies used in this study showed a high level of immune cell infiltration.

**FIGURE 1:**
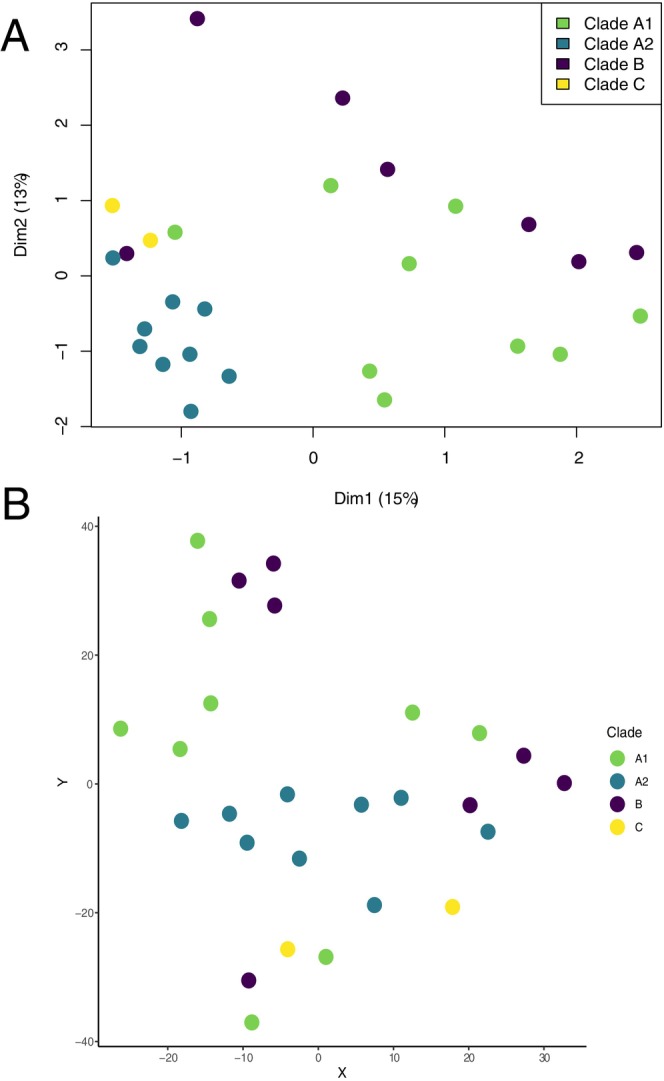
(A) MDS plot and (B) t‐SNE plot of distances between TMM normalized gene counts for each DFT1 sample. Neither method of dimension reduction indicated strong separation between the clusters. Clade A2 exhibited the tightest clustering of all groups, but this did not include all samples in A2. Samples are colored by genotypic clade.

**FIGURE 2:**
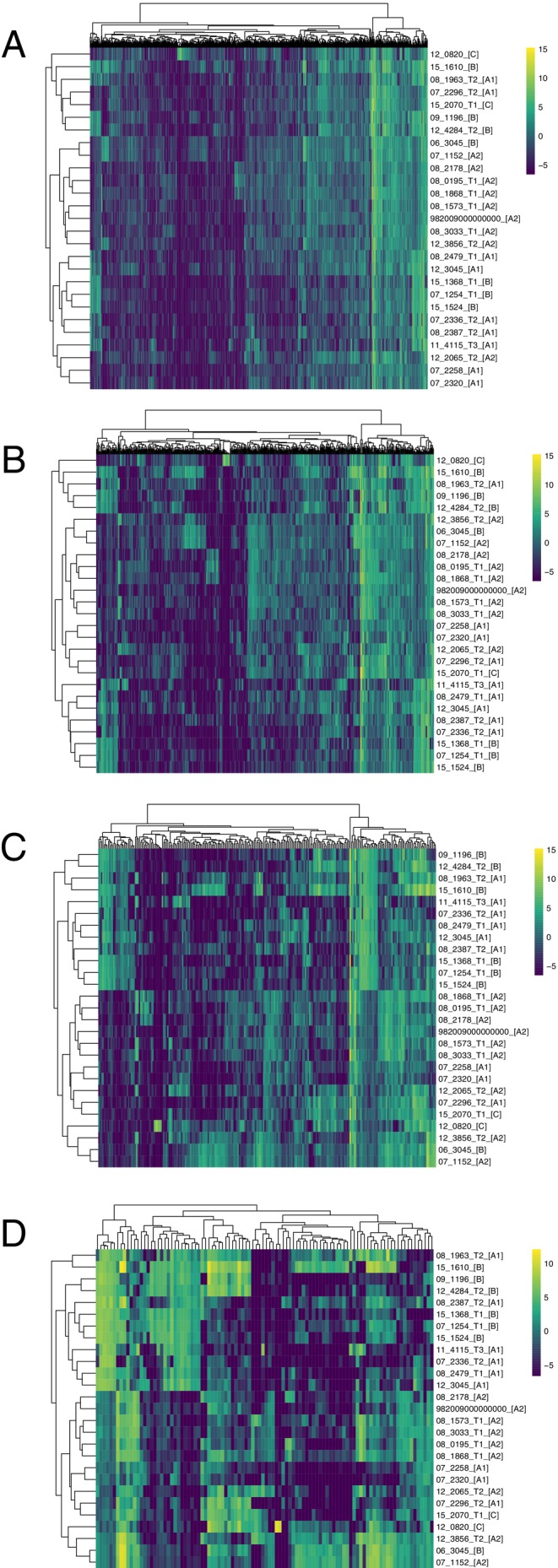
Heatmaps displaying (A) 1000 most variable genes (B) 500 most variable genes (C) 250 most variable genes (D) 100 most variable genes. None showed a clear mosaic pattern that would be expected if distinct clusters were present in the dataset. Yellow represents a higher value (indicating genes are upregulated in that sample) and dark blue represents a lower value (indicating that genes are downregulated in that sample). The name of the clade is in square brackets after the sample name.

Neither MDS (Figure 1A) nor t‐SNE (Figure 1B) suggested any underlying pattern in our DFT1 dataset. If distinct phenotypic subsets existed, we would expect to see datapoints with similar gene expression clustering together. Clade A2 exhibited the tightest clustering of all groups in the MDS plot (Figure 1A), however one Clade A2 sample showed tighter clustering with a Clade B sample. Clade A2 also did not exhibit tight clustering in t‐SNE (Figure 1B), indicating a lack of consistency. Similarly, none of the heatmaps show the type of clear mosaic pattern associated with distinct phenotypic groups, even when the data were reduced to the 100 most variable genes (Figure 2).

**FIGURE 3:**
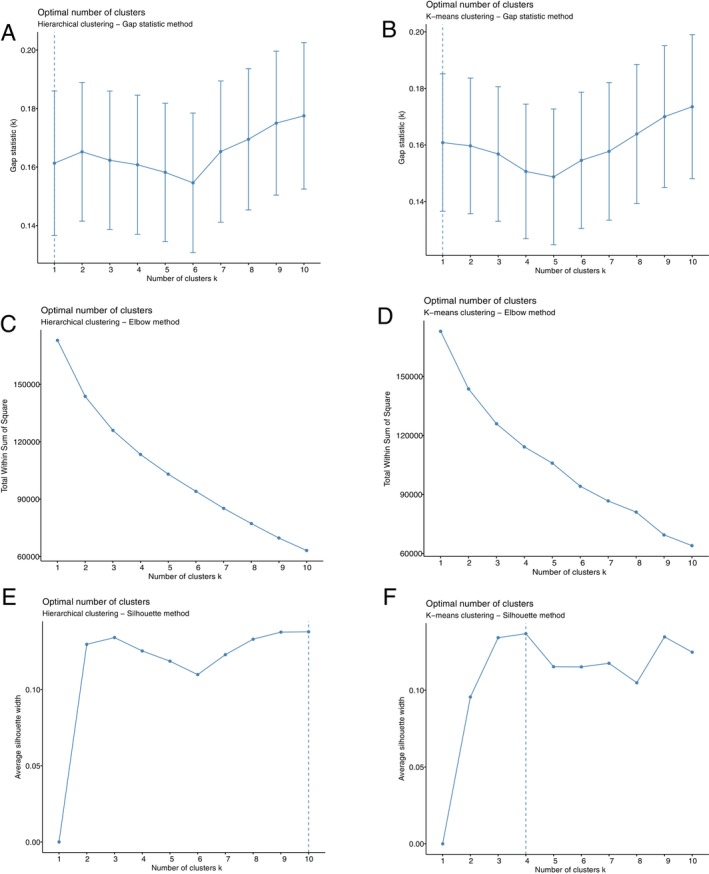
Clustering validation indices. The Gap statistic method indicated the optimum number of clusters was one for both (A) hierarchical clustering and (B) k‐means clustering; the Elbow method was ambiguous for both (C) hierarchical clustering and (D) k‐means clustering; and the Silhouette method indicated the optimum number of clusters was 10 (the maximum) for (E) hierarchical clustering and 4 for (F) k‐means clustering. The dotted line represents the highest value, that is, what the test deems to be the optimum number of clusters in the dataset.

**FIGURE 4:**
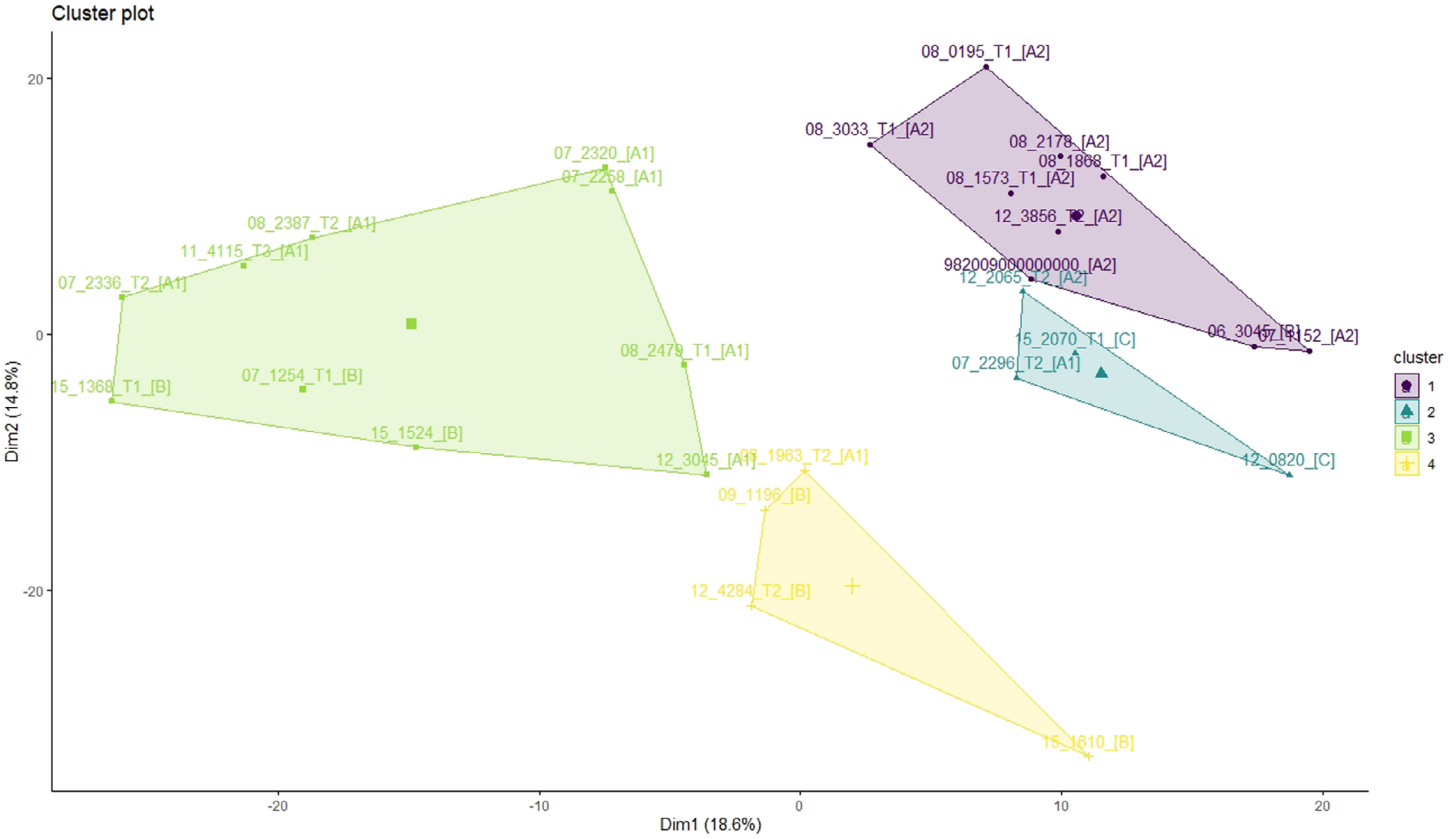
K means clustering results with *k* = 4. Each cluster contained samples from at least two different genotypic clades.

The lack of phenotypic subgroups amongst the DFT1 tumor sequences was further confirmed by the lack of a clear consensus between the hierarchical and k‐means clustering on the number of clusters within the dataset. However, the three validation indices (Gap, elbow, and Silhouette) generated contradictory values for k when applied to both hierarchical and k‐means clustering. The Gap statistic indicated the dataset contained no clusters (Figure 3A,B), the elbow method results were ambiguous (Figure 3C,D), and the Silhouette method indicated that the optimum number of clusters was 10 (the maximum) when applied to hierarchical clustering but *k* = 4 for k‐means clustering (Figure 3E,F). When k‐means clustering was performed with *k* set at 4 (Figure 4A), each cluster contained samples from at least two different clades.

Bootstrap analysis and Monte Carlo simulation of the hierarchical clustering proved similarly contradictory, with neither method completely supporting each other or any of the validation indices. The dendrogram generated from the bootstrapping analysis did not yield high values, with 11 out of 27 samples unable to be assigned to any clade with AU > 95 (a common cutoff value for determining significance) (Figure S6). The AU values are approximately unbiased probability values, which are obtained by multiscale bootstrap resampling and are more reliable than ordinary bootstrap resampling. Of the seven clusters that did have AU > 95%, six consisted of only two samples. The Monte Carlo‐based approach using sigclust2 identified four different significant nodes across the dendrogram (Figure S7).

The results of the clustering analysis on the full dataset (when not filtered for purity) are reported in Figures S8–S11.

## 4 Discussion

In this study, we demonstrate that there are no significant differences in gene expression patterns between Devil Facial Tumors from different genotypic subgroups. The lack of consensus between clustering methods and the lack of natural groups in the datasets using the visualization techniques (MDS, t‐SNE, heatmaps) indicated a single cluster for our dataset. This was further supported by the only validation index that constitutes a formal statistical test (the Gap method) (Patel et al. 2022). These results were consistent when applied to both the dataset filtered by > 80% purity (Figures 1, 2, 3, 4) and the full dataset (Figures S8–S11). Collectively, this shows that despite the genetic (Kwon et al. 2020) and karyotypic (Pearse et al. 2012) variation that exists between individuals, gene expression remains largely consistent across a broad temporal range (2006–2015) and geographic range (~21,120 km^2^).

Vaccination has been successful in managing other wildlife diseases, such as rabies (MacInnes et al. 2001; Slate et al. 2009), but never for cancers. Cancer is highly heterogeneous, often consisting of multiple molecular subtypes. A common method of defining these subtypes of cancer is through transcriptional profiling (Wang et al. 2022; Laurell et al. 2009; Gao, Qiu, et al. 2017; Kunz et al. 2018; Lehmann et al. 2011)—identifying groups with similar patterns of gene expression to guide treatment. Our results indicate that such transcriptomic subtypes are not present in DFT1, and so a single vaccine does have the potential to be effective for a large cross‐section of tumors.

Typically, we would expect to see variation among tumor biopsies in such a clustering analysis. This is because most tumors arise in different individuals and have a different underlying genetic profile. DFT1 is different, being a clonal cell line that has only arisen once in a single female Tasmanian devil (Murchison et al. 2010). As a transmissible cancer, we were not sure what we would see as this single cell has passed from animal to animal and undergone many cell divisions since it evolved at least 30 years ago. As such, we were surprised to see such a stable phenotype. However, this does appear to be consistent with previous studies that have implied DFTD has an unusual genetic stability for a cancer.

In addition to variation between individuals, non‐contagious cancers also exhibit a high level of heterogeneity within individuals. Many tumors are composed of subclonal populations that may be considered analogous to the clades of DFT1, as both have arisen from a single cell (Morris et al. 2016). Unlike DFT1, these subclones often exhibit phenotypic diversity. High levels of heterogeneity are associated with poor prognosis due to the selective pressure of chemotherapy and other treatments (Gao, Kim, et al. 2017; Greaves and Maley 2012).

However, DFT1 is also unusual as it is not treated with chemotherapy, and devils rarely mount an immune response against the disease (Pye, Hamede, et al. 2016). DFT1 cells do not express MHC‐I molecules, so they are not recognized by the host immune system (Siddle et al. 2013). This susceptibility to disease is likely exacerbated by low immune gene diversity in the Tasmanian devil (Morris et al. 2013). We suggest that this lack of immune response weakens the co‐evolutionary arms race between the host and pathogen, resulting in a more stable phenotype.

DFT1 also occupies an extremely narrow ecological niche for a contagious disease, being one of the few pathogens that are simultaneously an infectious agent and a mammalian cell (Metzger and Goff 2016). If deviations beyond a particular phenotype result in unviable or non‐transmissible cells (Pearse et al. 2012), there is likely a strong selective pressure to evolve slowly. Such a conserved phenotype has also been observed in the only other contagious cancer to afflict vertebrates (Canine Transmissible Venereal Tumor, CTVT) with histopathological screening showing no significant histopathological differences between clades (Strakova 2017). Although transcriptomics has been used in CTVT (Frampton et al. 2018), this study focused on the differential response to treatment rather than natural phenotypic variation between samples.

One previous study has suggested that transcriptomic differences exist in DFT1 tumors from different geographic locations (Kozakiewicz et al. 2021). However, we also suggest that pairwise comparisons between locations are not informative, as they assume each location only contains a single subtype, with no migration occurring between sites. Neutral population genetic data contradict this however, showing movement across the landscape in Tasmania (Farquharson et al. 2022).

We acknowledge that our results should be interpreted in the context of the study's limitations, namely that methods for estimating tumor purity were restricted due to the nature of the data. However, this study and dataset both represent a substantial contribution to the field of DFTD research and improve on previous studies that did not estimate tumor purity (Kozakiewicz et al. 2021).

In conclusion, our results show that DFT1 does not consist of multiple transcriptomic subtypes and that a one‐shot vaccine that will work across all clades should have the potential to manage the disease. As one of the largest studies to use transcriptomics to investigate phenotypic variation within DFT1, it also provides novel insights into this unique group of diseases.

## Disclosure

Benefit‐sharing statement: This study complies with benefit sharing under the Convention on Biological Diversity.

## Conflicts of Interest

The authors declare no conflicts of interest.

## Supporting information


Data S1.

Data S2.

Data S3.

## Data Availability

The raw sequencing reads generated and analysed during this study are available in the National Centre for Biotechnology Information (NCBI) short read archive under BioProject number PRJNA1067341.
