# Supplementary material for: No Evidence for Distinct Transcriptomic Subgroups of Devil Facial Tumor Disease (DFTD)
Source: Evol Appl. 2025 Apr 1;18(4):e70091. doi: 10.1111/eva.70091 (PMC11961399; doi:10.1111/eva.70091)
Supplement: Supplementary file 1 — Data S1. [file EVA-18-e70091-s003.docx]

**Supplementary Material**

**Supplementary table 1**. Metadata for 35 DFT1 samples used in this study. Phylogenomic clade was based on the analysis conducted by Kwon et al^1^. Strain was determined by cytogenetic analysis conducted by Kwon et al^1^.

| **Sample_Name** | **Transponder** | **Year sampled** | **Location sampled** | **Clade** | **Strain** |
| --- | --- | --- | --- | --- | --- |
| 07_2320 | 982009100758259 | 2007 | Sorell | Clade A1 | Strain 3 |
| 07_2296_T2 | 982009102737669 | 2007 | Sorell | Clade A1 | Strain 3 |
| 07_2297 | 982009100322308 | 2007 | Sorell | Clade A1 | Strain 3 |
| 07_2336_T2 | 985120015992583 | 2007 | Sorell | Clade A1 | Strain 3 |
| 08_1451_T2 | 982009104928332 | 2008 | Kempton | Clade B | Strain 2 |
| 08_1963_T2 | 982009105188074 | 2008 | Coles Bay | Clade A1 | No data |
| 08_1573_T1 | 982009104855230 | 2008 | West Pencil Pine | Clade A2 | Strain 2 |
| 08_0195_T1 | 982009104859904 | 2008 | Narawntapu | Clade A2 | Strain 1 tetraploid |
| 08_2387_T2 | 982009105171583 | 2008 | Sorell | Clade A1 | Strain 3 evolved |
| 08_2479_T1 | 982009100799027 | 2008 | Sorell | Clade A1 | No data |
| 07_2258 | 982009100754517 | 2007 | Sorell | Clade A1 | Strain 3 |
| 11_3909 | 982000123133145 | 2011 | Bronte | Clade B | No data |
| 11_3918 | 982000123216973 | 2011 | Bronte | Clade B | Strain 2 evolved |
| 12_2065_T2 | 982009104841875 | 2012 | West Pencil Pine | Clade A2 | Strain 1 |
| 12_0705_T2 | 982009104253781 | 2012 | West Pencil Pine | Clade C | Strain 1 |
| 11_2749_T1 | 982009104785985 | 2011 | West Pencil Pine | Clade C | No data |
| 11_4115_T3 | 982009106218282 | 2011 | Mount Pleasant | Clade A1 | Strain 4 |
| 12_0820 | 982009104719592 | 2012 | West Pencil Pine | Clade C | Strain 1 |
| 06_3045 | 00065DC9C5 | 2006 | Trowunna | Clade B | Strain 2 |
| 07_1152 | 982009100876802 | 2007 | Coles Bay | Clade A2 | Strain 2 |
| 07_1254_T1 | 982009102235882 | 2007 | Fentonbury | Clade B | Strain 2 |
| 08_1868_T1 | 985120016082881 | 2008 | Narawntapu | Clade A2 | Strain 6a |
| 08_2178 | 982009104358247 | 2008 | Ringarooma | Clade A2 | Strain 2 evolved |
| 08_3033_T1 | 982009102236127 | 2008 | Narawntapu | Clade A2 | Strain 1 diploid and tetraploid |
| 08_3696 | 982009104798550 | 2008 | Sorell | Clade A1 | No data |
| 09_1196 | 00065D7B0B | 2009 | Trowunna | Clade B | No data |
| 12_3045 | 982000123208272 | 2012 | DPIPWE (x Forestier) | Clade A1 | Strain 3 |
| 15_1368_T1 | 982009106485186 | 2015 | Narawntapu | Clade B | No data |
| 15_1524 | 982000191009681 | 2015 | Fentonbury | Clade B | No data |
| 15_1610 | 982000363454290 | 2015 | Fentonbury | Clade B | No data |
| 15_2070_T1 | 982009106575584 | 2015 | Takone | Clade C | No data |
| 982009000000000 | 982009106207596 | Unknown | Takone | Clade A2 | No data |
| 12_3856_T2 | 982000167789148 | 2012 | West Pencil Pine | Clade A2 | Strain 1 |
| 12_4284_T2 | 982009106180793 | 2012 | West Pencil Pine | Clade B | Strain 2 |
| 12_4284_T3 | 982009106180793 | 2012 | West Pencil Pine | Clade A2 | Strain 2 |

**Supplementary Table 2.** RDA and variance partitioning analysis results. The full model (Gene count ~ study + tissue type) had an adjusted R^2^=0.61, indicating that it accounts for ~61% of the variation in gene counts. Tissue type was significant as a predictor variable (p=0.001), but study was not (p=0.186). Variance partitioning analysis showed tissue alone (while controlling for the effect of study) accounted for 31% of the variation in gene count (p=0.001) and study (while controlling for the effect of tissue) only accounted for 0.7%. The ANOVA on the variance partitioning analysis also showed that study was not statistically significant (p=0.2).

|  | **Adjust R^2^** | **df** | **Variance** | **F** | **p-value** |
| --- | --- | --- | --- | --- | --- |
| ***RDA*** |  |  |  |  |  |
| Gene count ~ study + tissue type | 0.61 | 10 | 39971 | 9.2501 | 0.001* |
| Tissue | - | 8 | 38750 | 11.2094 | 0.001* |
| Study | - | 2 | 1221 | 1.4129 | 0.186 |
| Residual | - | 43 | 18581 | - | - |
|  |  |  |  |  |  |
| ***Variance partitioning analysis*** |  |  |  |  |  |
| [a] = Tissue \| Study | 0.31 | 8 | 20908 | 6.0481 | 0.001* |
| [b] = Study \| Tissue | 0.007 | 2 | 1221 | 1.4129 | 0.2 |
| [c] = Tissue + Study | 0.29 | 0 | - | - | - |
| [d] = Residuals | 0.39 | - | - | - | - |


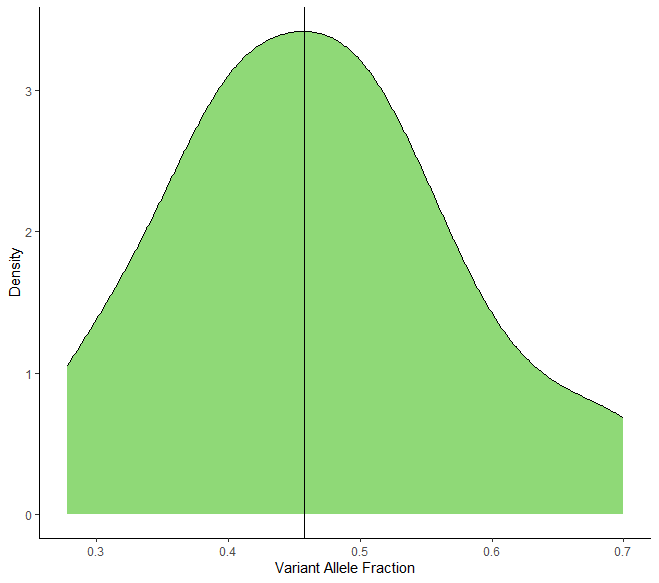


**Supplementary Figure 1**. VAF distribution plot for sample 982009000000000. The vertical line is at the maximum density of the distribution and represents VAF_HET_ (the mode VAF of heterozygous variants). In this example, VAF_HET_= 0.46 and so tumour purity ($\rho$) for this sample has been estimated to be 0.92 (ie 2*0.46).

**
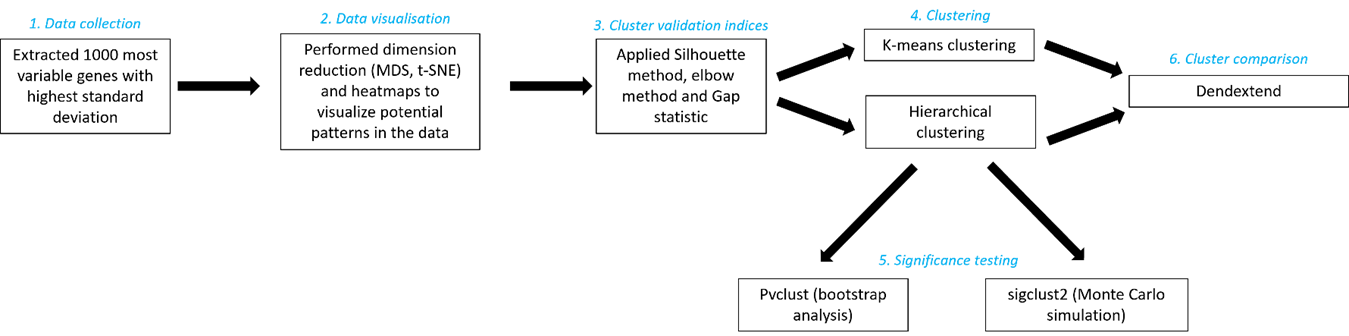
**

**Supplementary Figure 2.** Experimental design.


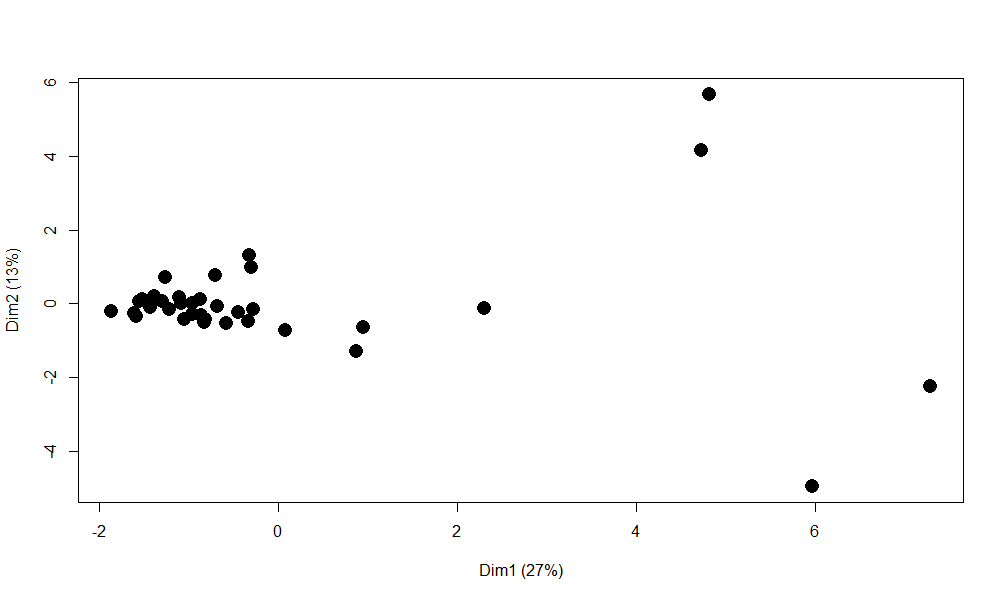


**Supplementary Figure 2.** MDS plot of all 35 DFT1 samples. This plot revealed two pairs of outliers that were separated from the rest of the samples on both Dimension 1 and Dimension 2, as indicated by the red circle


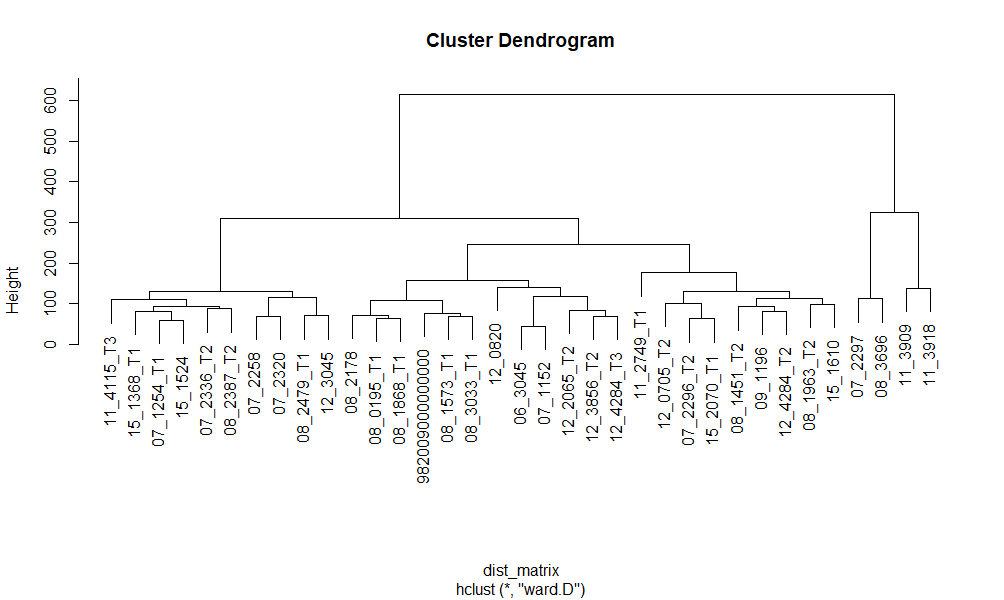


**Supplementary Figure 3.** Hierarchical clustering of all 35 DFT1 samples using Ward’s D and Euclidean distance. The same four outliers in figure 1a were assigned to their own cluster (red box), distinct from the other samples.

**
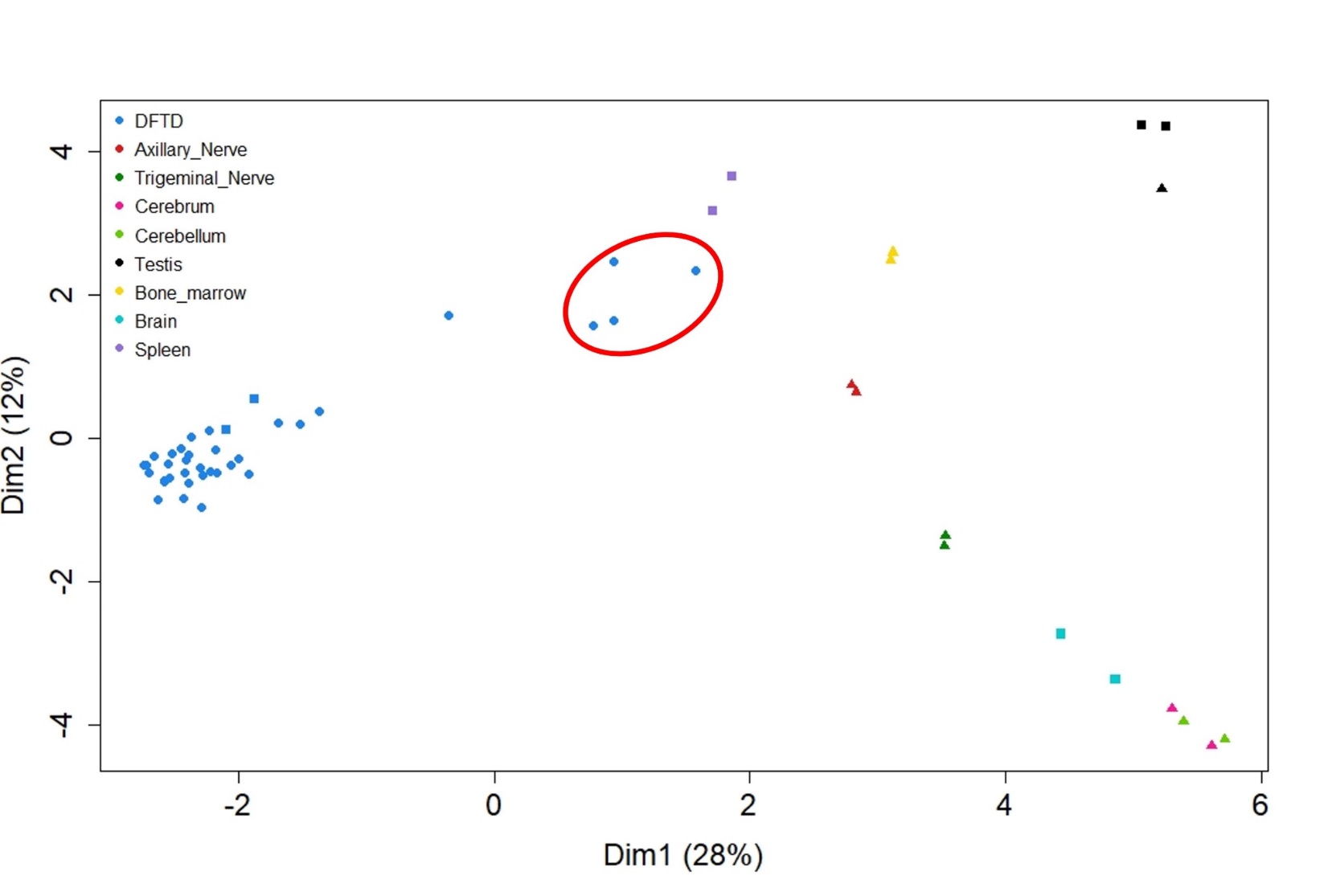
**

**Supplementary Figure 4**. MDS plot of the entire dataset (n = 37 DFT1 biopsies, two axillary nerve^[29]^, two bone marrow^[29]^, two brain^[35]^, two cerebellum^[29]^, two cerebrum^[40]^, two spleen^[35]^, three testes^[29,35]^ and two trigeminal nerve). Samples from the current study are represented by circles, samples from Patchett (2020) are represented by squares and samples from Stammnitz (2023) are represented by triangles. The four samples identified as outliers in figure 1a and 1b clustered more closely to the healthy tissue biopsies than the DFT1 biopsies (circled).


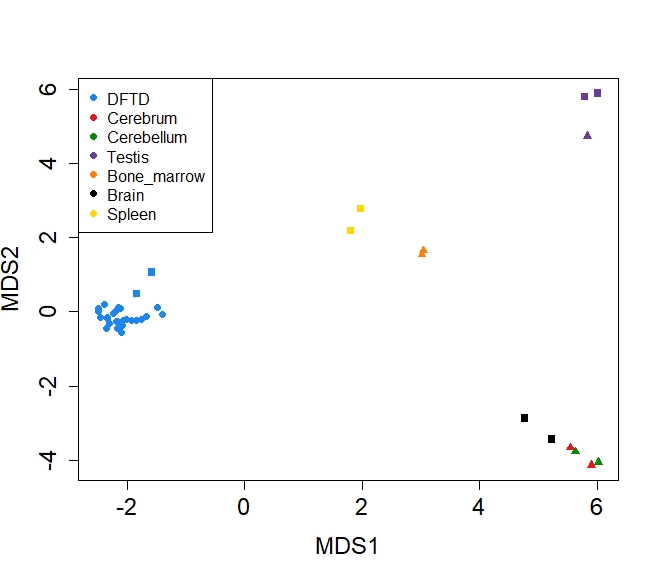


**Supplementary Figure 5**. MDS plot of 29 DFT1 samples and 17 healthy tissue samples, excluding the samples with purity < 80%. Samples from the current study are represented by circles, samples from Patchett (2020) are represented by squares and samples from Stammnitz (2023) are represented by triangles


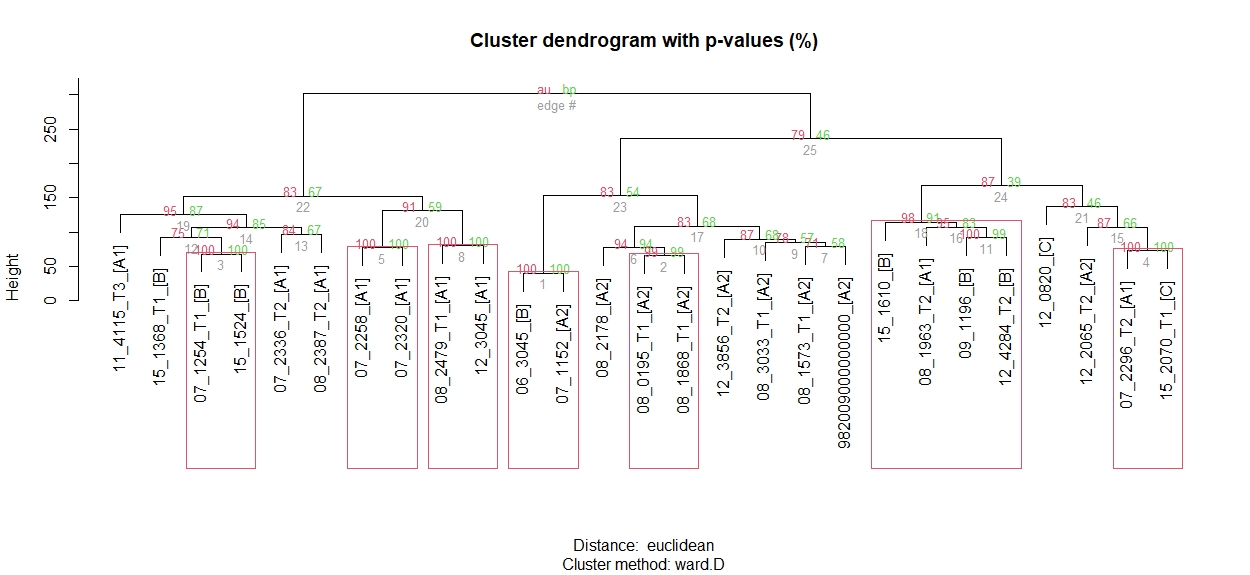


**Supplementary Figure 6.** Results from significance testing of hierarchical clustering results using pvclust. Values in red are AU (unbiased probability values obtained by multiscale bootstrap resampling), values in green are BP (obtained by ordinary bootstrap resampling). Clusters with BP > 95 are contained in a red rectangle. AU values are low across most of the dendrogram, with a large portion unable to be assigned to any clade.


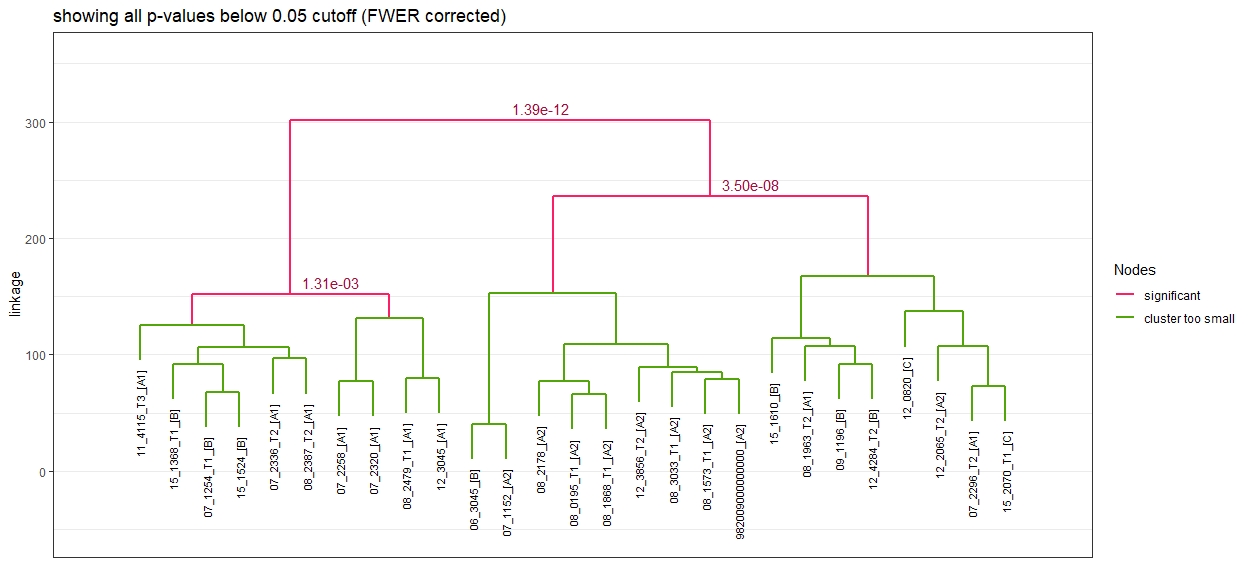


**Supplementary Figure 7.** Statistical significance of hierarchical testing results using Monte-Carlo based method with sigclust2. Four nodes are statistically significant and the clusters within these are too small for significance testing.


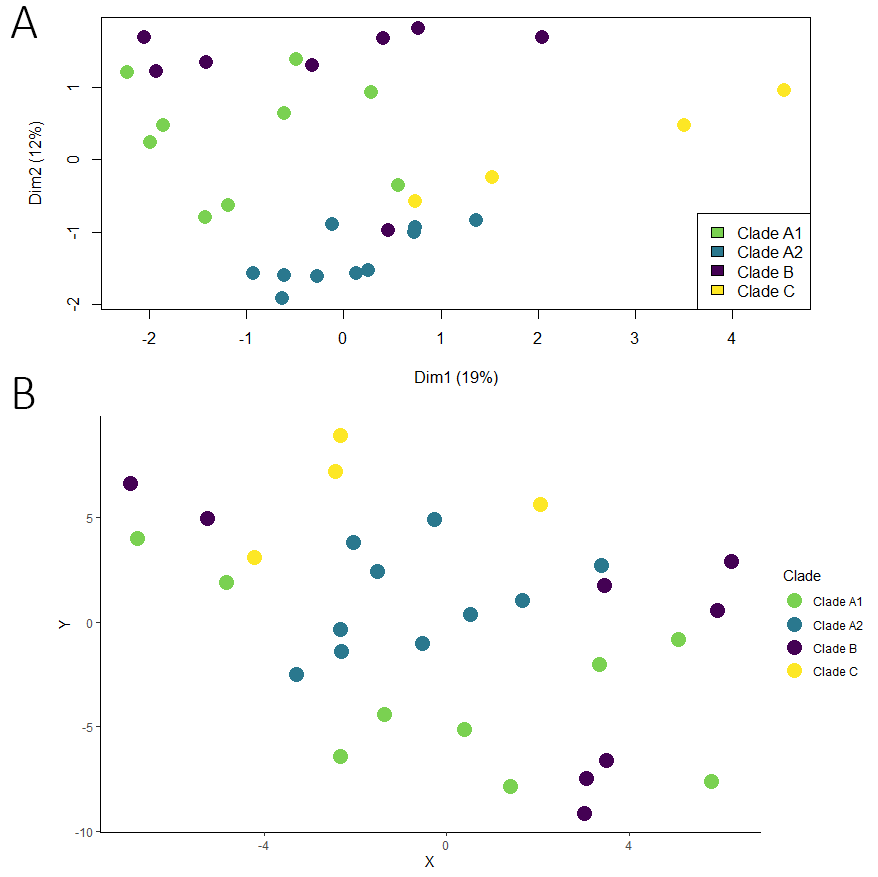


**Supplementary Figure 8**. (A) MDS plot and (B) t-SNE plot of distances between TMM normalised gene counts for each DFT1 sample when not filtered for purity. Neither method of dimension reduction suggested the presence of natural groups in the data, with all samples largely clustering together. Samples are coloured by genotypic clade.


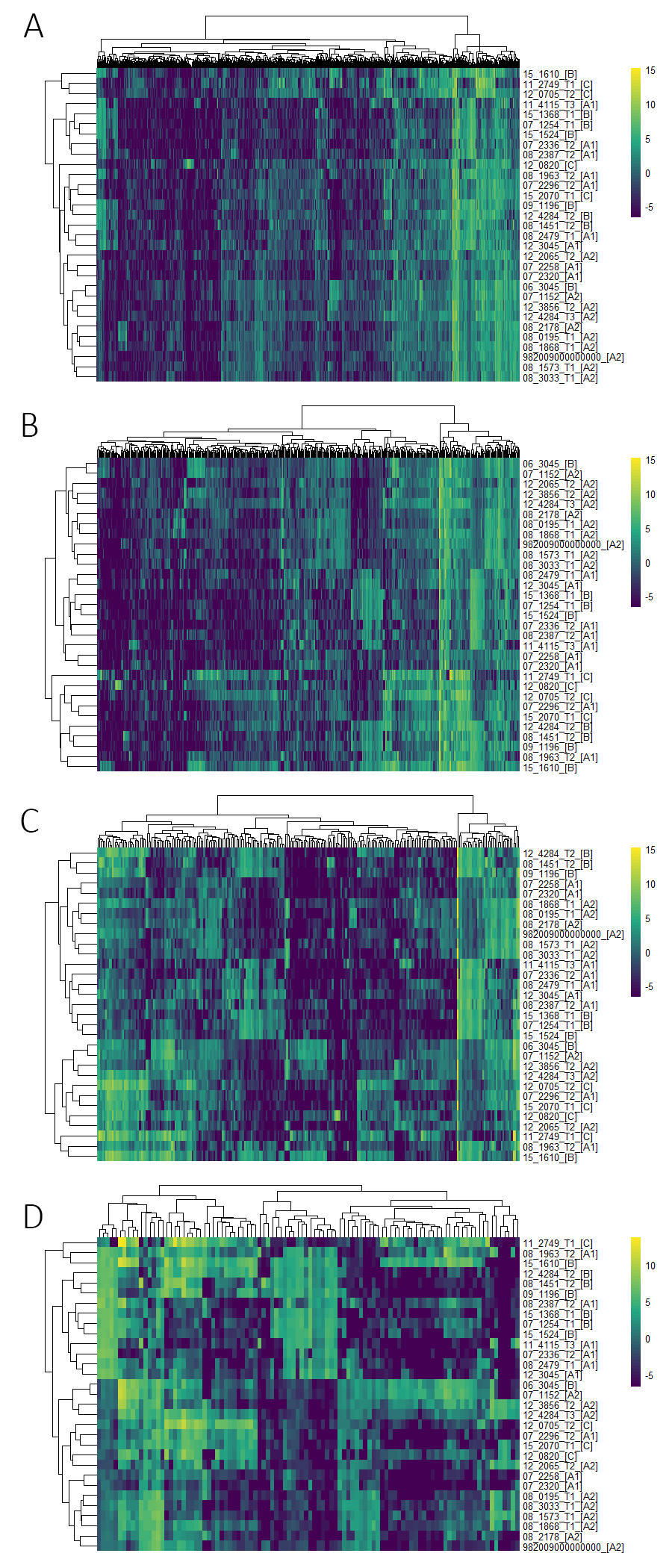


**Supplementary Figure 9**. Heatmaps displaying (A) 1000 most variable genes (B) 500 most variable genes (C) 250 most variable genes (D) 100 most variable genes of all samples (not filtered for purity). None showed a clear mosaic pattern that would be expected if distinct clusters were present in the dataset. Yellow represents a higher value (indicating genes are upregulated in that sample) and dark blue represents a lower value (indicating that genes are downregulated in that sample). The name of the clade is in square brackets after the sample name.


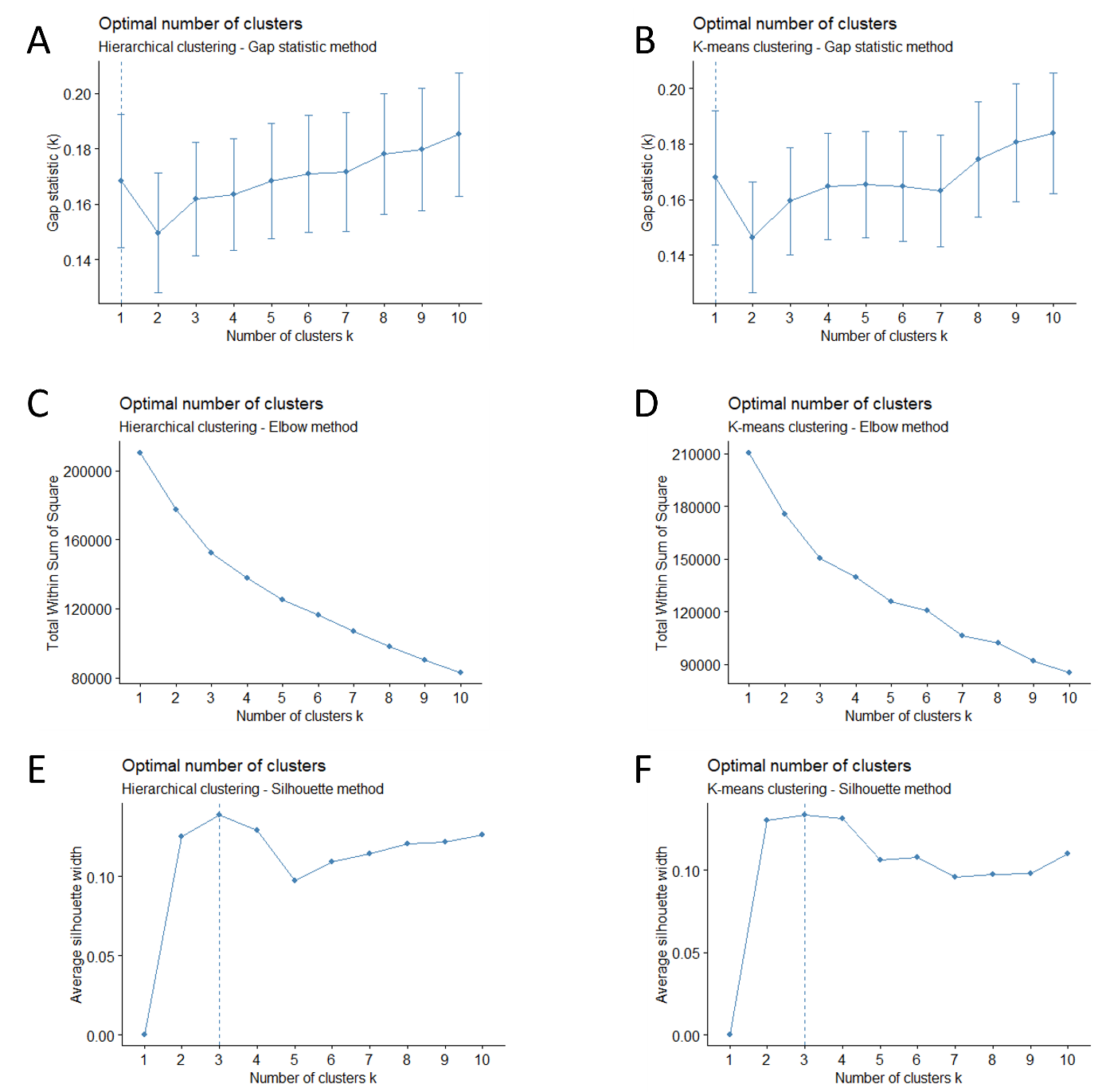


**Supplementary Figure 10.** Clustering validation indices on the full dataset (when not filtered for purity). Gap statistic method indicated the optimum number of clusters was one for both (A) hierarchical clustering and (B) k-means clustering; the Elbow method was ambiguous for both (C) hierarchical clustering and (D) k-means clustering; and the Silhouette method indicated the optimum number of clusters was three for both (E) hierarchical clustering and (F) k-means clustering. The dotted line represents the highest value i.e. what the test deems to be the optimum number of clusters in the dataset.


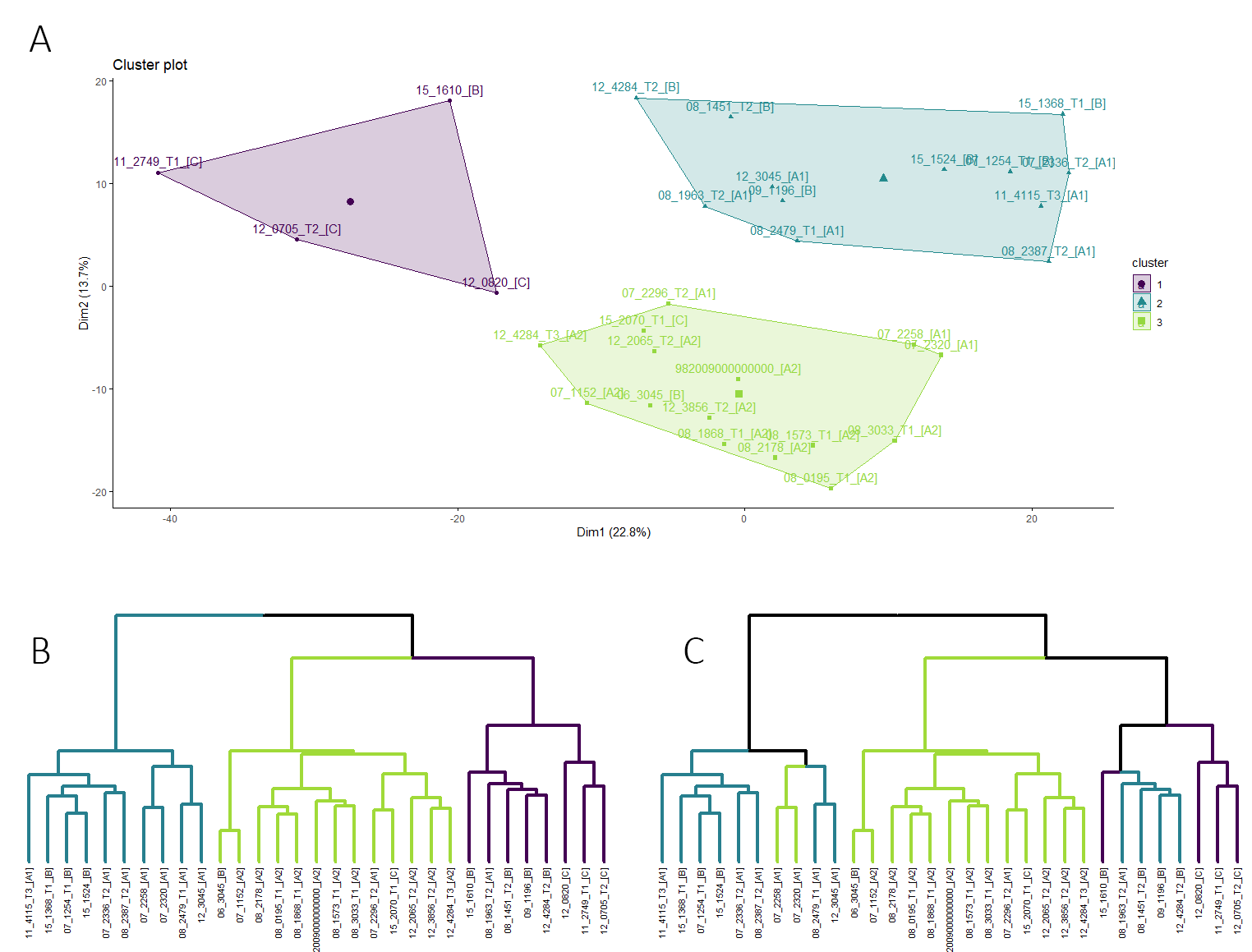


**Supplementary Figure 11**. Clustering results on the full dataset when not filtered for purity: (A) K-means clustering results with k=3. (B) Dendrogram depicting the results of hierarchical clustering. The samples have been coloured to according to which group the hierarchical clustering assigned them. (C) Dendrogram depicting the results of hierarchical clustering. For comparison, the samples have been coloured to according to which group the k-means clustering assigned them. The clade is in square brackets after the sample name. This indicates that clusters defined by the two different methods overlapped but did not reach a consistent consensus.

**References**

1 Kwon, Y. M. *et al.* Evolution and lineage dynamics of a transmissible cancer in Tasmanian devils. *PLoS biology* **18**, e3000926 (2020).
