# Supplementary figures and images for: No Evidence for Distinct Transcriptomic Subgroups of Devil Facial Tumor Disease (DFTD)
Source: Evol Appl. 2025 Apr 1;18(4):e70091. doi: 10.1111/eva.70091 (PMC11961399; doi:10.1111/eva.70091)

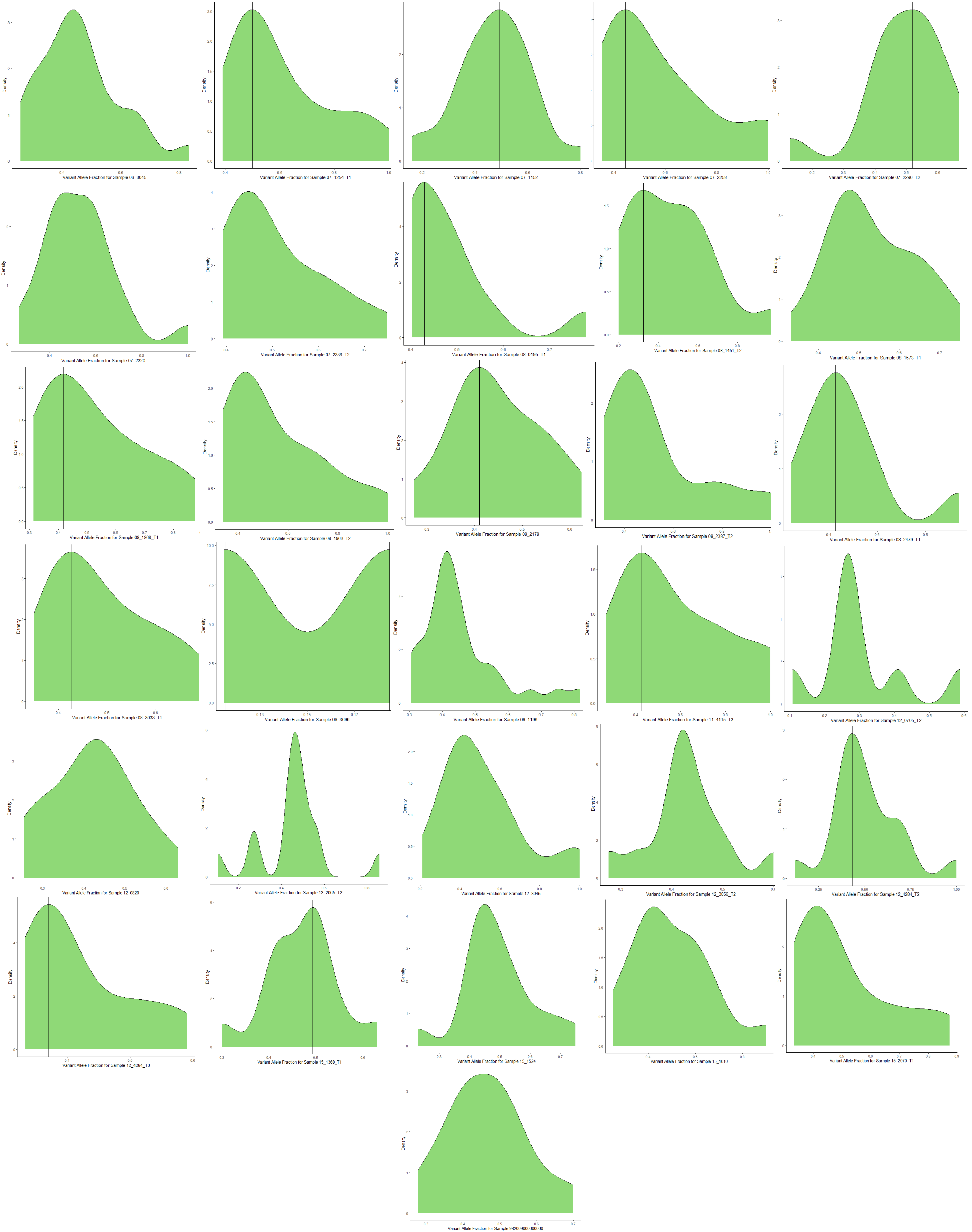

**Supplementary File 2.** VAF distributions for all samples with 2 or more trunk variants

Supplement: Supplementary file 2 — Data S2. [file EVA-18-e70091-s002.pdf]
